# The Effect of Metformin on Pituitary Function in Postmenopausal Women with Subclinical Hypothyroidism and Macroprolactinemia: A Single-Center Prospective Case–Control Study

**DOI:** 10.3390/ph18060834

**Published:** 2025-06-02

**Authors:** Robert Krysiak, Witold Szkróbka, Karolina Kowalcze, Bogusław Okopień

**Affiliations:** 1 Department of Internal Medicine and Clinical Pharmacology, Medical University of Silesia, 40-752 Katowice, Poland; rkrysiak@sum.edu.pl (R.K.); bokopien@sum.edu.pl (B.O.); 2 Department of Pediatrics in Bytom, School of Health Sciences in Katowice, Medical University of Silesia, Stefana Batorego 15, 41-902 Bytom, Poland; kkowalcze@sum.edu.pl; 3 Department of Pathophysiology, Faculty of Medicine, Academy of Silesia, Rolna 43, 40-555 Katowice, Poland

**Keywords:** anterior pituitary cells, glucose homeostasis, macroprolactin, postmenopause, secretory function, thyroid hypofunction

## Abstract

**Background/Objectives:** Metformin inhibits secretory function of overactive thyrotrophs, gonadotrophs, and lactotrophs. The clinical significance of an excess of high-molecular-weight prolactin (macroprolactinemia) remains unclear. The aim of the current study was to investigate for the first time whether macroprolactinemia determines the pituitary effects of this drug. **Methods:** This single-center prospective case–control study included two groups of postmenopausal women with subclinical hypothyroidism, who were matched for age, insulin sensitivity, and plasma concentrations of gonadotropins and TSH. Group A enrolled women with normal prolactin status, while group B included women with macroprolactinemia. Owing to concomitant type 2 diabetes or prediabetes, all the participants received metformin for six months. The outcomes of interest included glucose homeostasis markers (fasting glucose, glycated hemoglobin, and HOMA-IR), plasma prolactin (total and monomeric), macroprolactin, other pituitary hormones (FSH, LH, TSH, and ACTH), and peripheral hormones (estradiol, free thyroid hormones, and IGF-1). **Results:** Before metformin treatment, the study groups differed only in concentrations of total prolactin and macroprolactin. Metformin decreased FSH and TSH and tended to decrease LH only in group A, and the strength of this effect showed correlations with the baseline levels of these hormones, the degree of improvement in insulin sensitivity, and the macroprolactin content (only in group B). The decrease in fasting glucose, glycated hemoglobin, and HOMA-IR was more pronounced in group A than group B. There were no differences between the pretreatment and posttreatment values of total prolactin, monomeric prolactin, macroprolactin, ACTH, estradiol, free thyroid hormones, and IGF-1. **Conclusions:** The obtained results suggest that macroprolactinemia may counteract the pituitary effects of metformin.

## 1. Introduction

Chronic metformin treatment results in the lowering of elevated levels of four anterior pituitary hormones: thyroid-stimulating hormone (TSH), follicle-stimulating hormone (FSH), luteinizing hormone (LH), and prolactin [1,2,3,4,5,6,7,8,9]. The pituitary effects of metformin seem to be associated with the accumulation of remarkable amounts of this drug in the pituitary tissue due to the lack of the blood–brain barrier [10,11]. An important finding concerning the pituitary effects of metformin is that the strength of its action is determined by the baseline secretion of each hormone. Because the drug does not affect hormone levels if they are within the reference range [1,2,4,8], metformin treatment does not seem to be complicated by pituitary hypofunction. From a clinical point of view, it is also important that the impact on pituitary hormones is observed independently of the reason for hormone excess [4,5,6], as well as that hormonal effects of metformin are more pronounced in individuals on high-dose metformin treatment [4,8].

According to the Endocrine Society, the term macroprolactinemia denotes a preponderance of high-molecular-weight forms of prolactin (macroprolactin) [12]. Macroprolactin, also known under the name of big-big prolactin, is, apart from monomeric prolactin or prolactin dimers, one of the three major isoforms of this hormone in circulation [12,13]. It is composed mainly of complexes of the monomeric hormone and immunoglobulins (mainly immunoglobulin G) and, though to a lesser extent, from covalent or noncovalent aggregates of the monomeric hormone [13,14]. Because of their large size, macroprolactin is characterized by long half-life, contributing significantly to an increase in total prolactin levels [15]. The prevalence of macroprolactinemia is estimated at 10–25% in individuals with hyperprolactinemia and at 3.7% in the general population [15]. Macroprolactin excess is considered to be significant if less than 40% of immunoreactive prolactin is monomeric and dimeric [16]. More subtle excess is a clinically insignificant laboratory deviation. In turn, an evident predominance of big-big prolactin may have either an asymptomatic course (biochemical macroprolactinemia) [17] or may lead to clinical manifestation (clinically relevant macroprolactinemia). In line with this distinction, an excess of big-big prolactin was found to be accompanied by symptoms similar to those observed in individuals with monomeric hyperprolactinemia, including oligomenorrhoea/amenorrhoea, galactorrhea, and subfertility/infertility, although they were reported less frequently than in monomeric hyperprolactinemia [18,19]. Moreover, long-term excess of big-big prolactin in women was found to be associated with reduced sexual desire [20] and increased levels of cardiometabolic risk factors [21]. Lastly, another interesting observation was that macroprolactinemia attenuated specific effects of other drugs in women: the impact of a statin on cardiometabolic risk [22] and the impact of levothyroxine on hypothalamic–pituitary–thyroid axis activity and thyroid autoimmunity [23].

**Figure 1:**
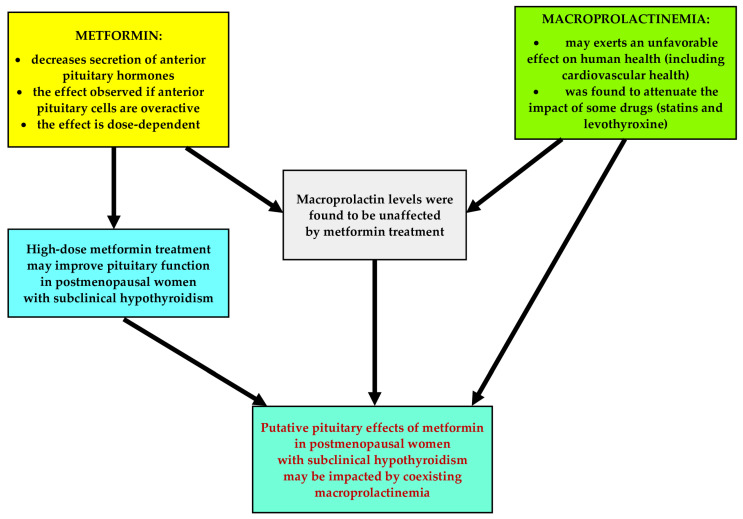
The research hypothesis.

There are some issues concerning the hormonal effects of metformin and macroprolactinemia that deserve better understanding. Firstly, it remains unanswered whether metformin affects pituitary hormone levels in the case of the overactivity of different populations of anterior pituitary cells. Secondly, because macroprolactinemia is associated with impaired insulin sensitivity [21], it is unknown whether the impact of metformin on glucose homeostasis markers is affected by an excess of high-molecular-weight complexes of prolactin. Thirdly, unlike total prolactin [6,7,8,9], metformin had a neutral effect on macroprolactin levels, but the study was underpowered, and 50% of the participants received metformin in a small daily dose (1.7 g) [24]. Lastly, considering that an excess of big-big prolactin counterbalanced the impact of statin therapy and levothyroxine replacement [22,23], it is possible that this condition is also one of the determinants of the pituitary effects of metformin. Thus, the aim of the current study was to investigate whether coexisting macroprolactinemia modulates the pituitary effects of metformin in postmenopausal women with subclinical hypothyroidism, who are characterized by a simultaneous elevation of TSH and gonadotropins levels. The research hypothesis is depicted in Figure 1.

## 2. Results

Before metformin treatment, both study groups were similar in terms of age, smoking habits, percentage of patients with prediabetes and type 2 diabetes, body mass index, and blood pressure (Table 1). The total percentage of women receiving other medications was 62% in group A and 58% in group B. The patients assigned to group A were treated with vitamin D supplements (34%), 3-hydroxy-3-methylglutaryl-CoA (27%), proton-pump inhibitors (23%), bisphosphonates (12%), allopurinol (8%), mebeverine (4%), and orlistat (4%). In turn, patients belonging to group B received vitamin D supplements (38%), proton-pump inhibitors (23%), 3-hydroxy-3-methylglutaryl-CoA (23%), bisphosphonates (15%), drotaverine (4%), febuxostat (4%), and tizanidine (4%). Plasma levels of total prolactin and macroprolactin were higher in group B than group A, while there were no between-group differences in fasting glucose, HbA_1c_, HOMA-IR, gonadotropins, TSH, monomeric prolactin, ACTH, estradiol, free thyroid hormones, and IGF-1 (Table 1). The levels of safety biochemical parameters were similar in both groups (Table 2).

Four women did not complete the study protocol. One patient in group A and two patients in group B were withdrawn because of adverse metformin effects (abdominal cramps, abdominal pain, diarrhea, and a metallic taste in the mouth), which resolved after treatment cessation. One woman from group A was lost to follow-up because the place of residence was changed. The remaining 48 patients completed the study, and none of them was classified as poorly compliant. There were no differences in the safety biochemical parameters before and after metformin treatment (Table 2). A post hoc power calculation showed adequate statistical power. The baseline values of the outcome variables were similar if only women who completed the study were analyzed.

**Figure 2:**
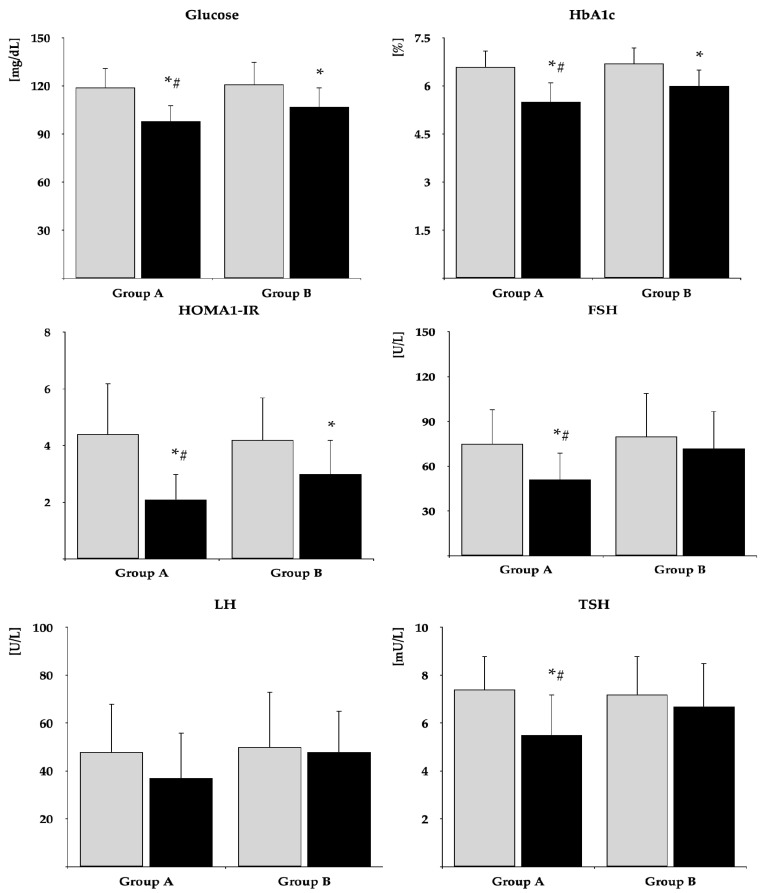
The impact of metformin on glucose homeostasis markers, gonadotropins, and TSH. Only women who completed the study were analyzed. The data are shown as the mean ± standard deviation. Group A: postmenopausal women with subclinical hypothyroidism and normal prolactin levels; group B: postmenopausal women with subclinical hypothyroidism and macroprolactinemia. Grey bars: before treatment; black bars: after treatment. * *p* < 0.05 vs. before treatment; ^#^ *p* < 0.05 vs. group B.

**Figure 3:**
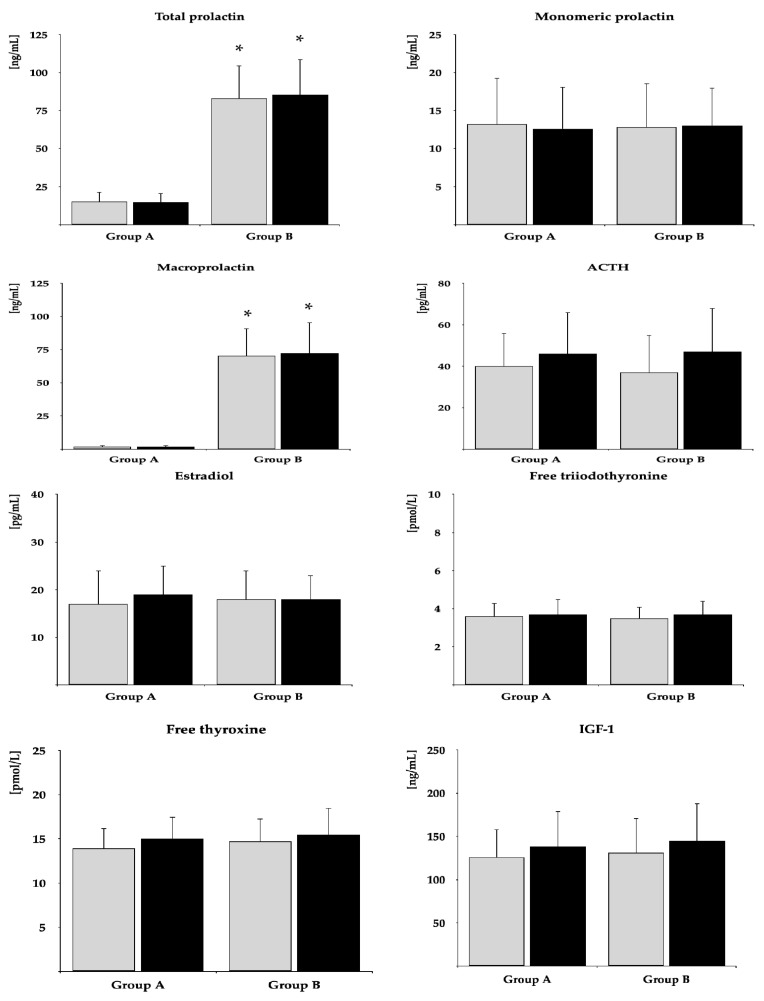
The impact of metformin on the remaining hormones. Only women who completed the study were analyzed. The data are shown as the mean ± standard deviation. Group A: postmenopausal women with subclinical hypothyroidism and normal prolactin levels; group B: postmenopausal women with subclinical hypothyroidism and macroprolactinemia. Grey bars: before treatment; black bars: after treatment; * *p* < 0.05 vs. group B.

**Figure 4:**
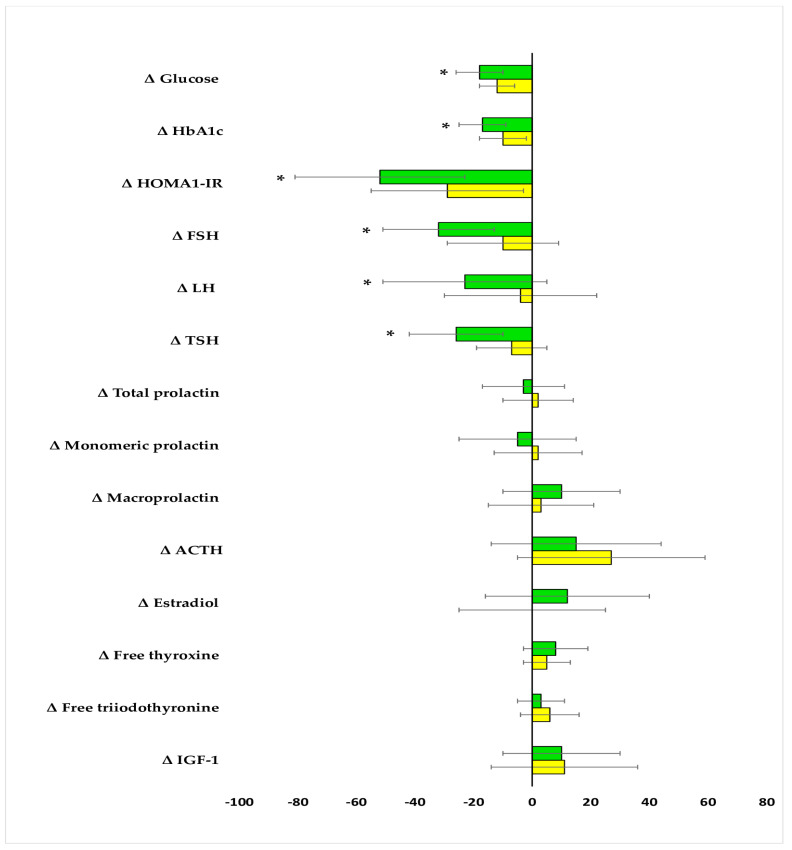
Percentage changes from baseline in the outcome variables in the study population. The data are shown as the mean ± standard deviation. Only women who completed the study were analyzed. Green bars: postmenopausal women with subclinical hypothyroidism and normal prolactin levels (group A); yellow bars: postmenopausal women with subclinical hypothyroidism and macroprolactinemia (group B); * *p* < 0.05 vs. group B.

The impact on fasting glucose, HbA_1c_, and HOMA-IR was observed in both groups. The drug reduced FSH and TSH and tended to reduce LH (*p* = 0.0569) only in group A. In neither group were there changes in total prolactin, monomeric prolactin, macroprolactin, ACTH, estradiol, free thyroxine, free triiodothyronine, and IGF-1 (Table 2). After six months of metformin treatment, fasting glucose, HbA_1c_, HOMA-IR, FSH, LH, and TSH were lower in group B than in group A. Posttreatment total prolactin and macroprolactin were higher in group B than group A (Figure 2 and Figure 3, Supplementary Table S1). The effect of metformin on fasting glucose, HbA_1c_, HOMA-IR, FSH, LH, and TSH was stronger in group A than group B (Figure 4, Supplementary Table S2). There were no differences between baseline and follow-up body mass index and blood pressure.

In both study groups, the effect of metformin on gonadotropins and TSH positively correlated with their baseline concentrations (FSH—group A: r = 0.605 [*p* < 0.0001], group B: r = 0.584 [*p* < 0.0001]; LH—group A: r = 0.528 [*p* < 0.0001], group B: r = 0.495 [*p* <0.0001]; TSH—group A: r = 0.612 [*p* < 0.0001], group B: r = 0.512 [*p* < 0.0001]). In group A, the impact of treatment on FSH positively correlated with the impact on LH (r = 0.319 [*p* = 0.0402]. There were also positive correlations between treatment-induced changes in gonadotropins and TSH and treatment-induced changes in HOMA-IR (FSH—group A: r = 0.432 [*p* = 0.0008], group B: r = 0.372 [*p* = 0.0065]; LH—group A: r = 0.395 [*p* = 0.0015], group B: r = 0.341 [*p* = 0.0286]; TSH—group A: r = 0.464 [*p* = 0.0002], group B: r = 0.298 [*p* = 0.0496]). In group B, the impact of treatment on gonadotropins inversely correlated with total prolactin (FSH: r = −0.416 [*p* = 0.0008]; LH: r = −0.358 [*p* = 0.0071]; TSH (r = −0.408 [*p* = 0.0010]) and macroprolactin (FSH: r = −0.476 [*p* = 0.0002]; LH: r = −0.424 [*p* = 0.0006]; TSH r = −0.487 [*p* = 0.0001]).

## 3. Discussion

Metformin administered to postmenopausal women with subclinical thyroid hypofunction and normal prolactin homeostasis reduced FSH and TSH levels and tended to decrease LH levels. More pronounced postmenopausal changes in FSH than LH explain why the metformin action reached the level of statistical significance only in case of the former hormone. Moreover, the degree of reduction in gonadotropins and TSH depended on the initial concentrations of these hormones but was unrelated to baseline secretions of other pituitary hormones. Moreover, no changes were observed in the case of monomeric prolactin, ACTH, and IGF-1. The last hormone mediates most of the biological effect of growth hormone and is used to assess its mean secretion (because of pulsatile secretion, growth hormone is undetectable in circulation for most of the day) [25]. These findings are further evidence that the pituitary effects of metformin are determined by the degree of hormone overproduction. Moreover, the study’s results suggest that treatment-induced changes in pituitary hormones produced by various populations of anterior pituitary cells are not reciprocally related. Although there were weak correlations between the impact on FSH and on LH, the decrease in TSH did not correlate with the changes in gonadotropins. Despite high-dose treatment, the effect of metformin on FSH and TSH was moderate, and the follow-up levels of these hormones were still higher than the reference values. As our study shows, high-dose metformin treatment was well tolerated by postmenopausal hypothyroid women (even by females with prediabetes), and only 4% of participants prematurely terminated the study because of adverse effects of metformin. However, considering that changes in pituitary hormones were not accompanied by changes in peripheral hormones (estradiol and free thyroid hormones), metformin treatment may bring more benefits in the prevention and treatment of pathologies directly resulting from FSH and TSH excess, including osteoporosis, dementia, TSH-secretion tumors, or goiter [26,27,28] in comparison to the treatment of menopausal symptoms and thyroid hypofunction.

The second important finding of our study was that metformin did not reduce macroprolactin levels, though these levels were markedly elevated, as well as that the impact of treatment on macroprolactin concentration did not correlate with the baseline concentration of big-big prolactin. Because the only difference between the study groups was macroprolactin content, our findings cannot be explained by the impact of comorbidities, cotreatments, differences in anthropometric parameters or differences in glucose homeostasis markers. This study’s finding is in line with our previous study, which included a group of only 12 women with isolated macroprolactinemia, the majority of whom were in reproductive age [24]. Similar results in young and postmenopausal women suggest that the neutral effect on macroprolactin is unrelated to age. It seems unlikely that our results are associated with variations in macroprolactin concentrations. Both groups of participants were recruited in similar proportions over the entire year. Moreover, although metformin-naive individuals with macroprolactinemia were not enrolled, the big-big prolactin levels in this condition were found to remain stably elevated for many months [29,30].

Some relevant conclusions can be inferred from these results. Firstly, monomeric prolactin is probably a more specific and sensitive marker of metformin action on prolactin production than the total hormone. To better assess the response to treatment, measurement of total prolactin should be replaced with measurement of the monomeric hormone. Secondly, the decrease in prolactin in patients in whom only total prolactin was measured [6,7,8,9] seems to have resulted from the impact on the monomeric hormone, even if prolactin was elevated due to an excess of both monomeric and high-molecular-weight prolactin. Thirdly, no changes in total prolactin in response to chronic metformin treatment may support the advisability of measuring macroprolactin, particularly in asymptomatic/oligosymptomatic patients. Fourthly, it is still unclear whether macroprolactinemia is a consequence of increased pituitary production or results from extra-pituitary production or impaired renal excretion. Although significant quantities of macroprolactin were detected in the pituitary gland [31], our results appear to favor the latter two possibilities (extrapituitary production or impaired renal clearance). Lastly, no differences in metformin action on macroprolactin in reproductive-age and postmenopausal women indicate that this effect is unrelated to the estrogen status of women. Previously, we observed that oral contraceptive pills containing ethinyl estradiol increased macroprolactin content [32]. Hence, the lack of metformin action on macroprolactin levels could have been explained by counterbalancing a direct inhibitory effect on macroprolactin by a stimulatory effect of endogenous estrogens. However, the current findings argue against this explanation.

There is some evidence suggesting that macroprolactinemia is associated with unfavorable changes in glucose homeostasis. Firstly, it was diagnosed more frequently in women with than without diabetes [33]. Secondly, individuals with diabetes were characterized by higher HbA_1c_ levels if they had a concomitant excess of macroprolactin [33]. Lastly, women with total prolactin levels exceeding 40 ng/mL and prolactin recovery below 40% had higher post-challenge plasma glucose and HOMA-IR than healthy women with macroprolactin levels within the reference range [21]. Thus, it may be assumed that a significant proportion of individuals with an excess of high-molecular-weight prolactin require antidiabetic treatment. However, postmenopausal women with macroprolactinemia were characterized by weak metabolic effects of this drug, which were less pronounced than in women with normal prolactin homeostasis. They suggest that metformin, at least when it is administered as a monotherapy, does not seem to be the drug of choice in the treatment of macroprolactinemic patients with type 2 diabetes and in the prevention of this disorder in high-risk patients with increased levels of high-molecular-weight prolactin. It is possible that such patients are better candidates for other groups of antidiabetic agents. Alternatively, women with macroprolactinemia and impaired glucose homeostasis may benefit from concomitant treatment with drugs reducing macroprolactin content. Unfortunately, as far as we know, only two therapeutic options have been found to decrease circulating levels of big-big prolactin, being potential candidates for the combination therapy with metformin: exogenous vitamin D [30] and dopamine agonists [18,34]. However, the impact of vitamin D was investigated only in young women, most of whom suffered from vitamin D deficiency [30], while the impact of dopaminergic agents was observed in some (but not in all) patients with macroprolactinemia [18,34]. Thus, their usefulness in combination therapy requires further investigation.

The clinical symptoms associated with an excess of big-big prolactin seem to be associated with the dissociation of high-molecular-weight prolactin complexes, particularly complexes of prolactin and immunoglobulin G, which are characterized by high capacity but low affinity [14]. The released monomeric hormone can then bind to the prolactin receptor, although direct stimulation of this receptor by big-big prolactin also cannot be totally ruled out [19,34]. The presence of inverse correlations between macroprolactin and the impact of treatment on gonadotropins and TSH indirectly suggests an interaction between metformin and macroprolactin at the level of target tissues. In particular, the potential target may be the pituitary gland, which is found to accumulate both metformin [10] and macroprolactin [31], though the underlying molecular mechanism remains elusive. There are several arguments in favor of the explanation that the most likely downstream pathway is AMP-activated protein kinase in anterior pituitary cells. Gonadotropin-secreting and TSH-secreting cells are two populations of pituitary cells with the highest expression of AMP-activated protein kinase [35]. This kinase plays a fundamental role in mediating metformin action on gonadotropin production, both baseline and stimulated [35]. Enhanced prolactin signaling was associated with diminished activity of AMP-activated protein kinase [36]. Lastly, the degree of reduction in FSH, LH, and TSH correlated with the improvement in insulin sensitivity, and the latter effect was proven to be mediated by stimulation of AMP-activated protein kinase [37].

The parallelism between differences in metformin action on gonadotropins and TSH and on insulin sensitivity may be also explained by the impact on tuberoinfundibular dopamine neurons, inhibiting prolactin release and down-regulated by prolactin [38,39]. Central dopamine agonists reduce glucose levels and insulin resistance [40]. There are also other arguments supporting this explanation. Metformin administered to women increased the central dopaminergic tone [41]. The strength of metformin action on TSH in women with polycystic ovary syndrome was determined by the activity of tuberoinfundibular dopamine neurons [42]. Dopamine agonists decreased LH release in women [43]. Lastly, dopamine suppresses TSH secretion, and this effect is mediated by D_2_ receptors [44].

The obtained results provide further evidence that biochemical macroprolactinemia cannot be considered as a laboratory artefact without clinical significance. The inhibitory effect of high concentrations of high-molecular-weight prolactin on the hormonal and metabolic effects of metformin is in line with its attenuating effect on the impacts of other drugs: atorvastatin [22] and levothyroxine [23], suggesting that this condition may worsen the effectiveness of different metabolic and hormonal therapies. It cannot be excluded that the clinical consequences of an excess of macroprolactin are more severe than those of monomeric hyperprolactinemia, the feature of which is rich clinical symptomatology, but also a good response to dopamine agonists [45]. In turn, despite the asymptomatic or oligosymptomatic course of macroprolactinemia in many patients, the impact of dopamine agonists and exogenous vitamin D in this condition is limited, and these agents only partially decrease (but do not normalize) macroprolactin content [18,30,34]. Thus, the obtained results are worth pursuing in further research.

### Advantages and Limitations of the Study

The major strength of the current study is its novelty. No previous study investigated the association between the macroprolactinemia and hormonal effects of metformin treatment. Our findings, together with previous evidence, suggest that macroprolactinemia may exert a multidirectional adverse effect on human health. The fact that the study population was relatively homogeneous helped us reduce bias resulting from the presence of confounding risk factors. The additional strengths of our study included its prospective nature, matching the study groups for factors that might have affected the obtained results, stable metformin dose, thorough monitoring of the patients’ compliance, and carrying out duplicate laboratory analyses. Considering the high prevalence of hypothyroidism and glucose homeostasis disorders, and the underestimation of macroprolactinemia prevalence, our findings may apply to a globally significant population of middle-aged or elderly women. Lastly, using the STROBE guidelines increased transparency and enhanced the credibility of our findings.

There are some study limitations that should also be borne in mind when interpreting our findings. Although the sample size exceeded the required minimum sample size, the number of participants was relatively small. The obtained results might have been impacted by systematic errors (selection biases, information biases, and confounding). Macroprolactin was separated using a precipitation method, while the gold standard method is currently gel filtration chromatography [46]. Owing to strict inclusion criteria, it is difficult to conclude whether macroprolactin also interacts with the pituitary effects of metformin in individuals with normal glucose homeostasis. Considering obligatory iodine prophylaxis and the low mean selenium intake by the subjects inhabiting the study area [47,48], the impact of macroprolactin on metformin action on the hypothalamic–pituitary thyroid axis does not have to be the same in iodine-deficient and selenium-sufficient populations. Lastly, we cannot completely rule out a late-appearing effect of non-pharmacological treatment.

## 4. Materials and Methods

### 4.1. Study Design and Setting

Our study was a single-center prospective case–control study carried out in the years 2018–2024 with a two-year break caused by the COVID-19 pandemic. The setting was a tertiary outpatient clinic, which was a referral center for patients with metabolic and endocrine disorders. The participants were initially cared for by local general practitioners, who cooperated with the members of our research team. The protocol conformed to the 1975 Declaration of Helsinki and its subsequent revisions and was approved by the institutional ethics review board. Written informed consent was obtained from all the patients after an explanation of the nature of the study and the possible consequences associated with participation. The study was reported in line with the STROBE reporting guidelines (Supplementary Table S3).

### 4.2. Participants

The study included 52 postmenopausal women aged 50 or older with type 2 diabetes or prediabetes and subclinical hypothyroidism. They were white Polish Caucasians living in the Upper Silesia Metropolis (a highly urbanized area in southern Poland, bordering Slovakia and the Czech Republic). All eligible patients adhered to the lifestyle modification for at least three months and were candidates for metformin treatment. Only patients who were not candidates for levothyroxine substitution were included. Women were considered postmenopausal if their last menstrual period had been more than twelve months before the onset of the study. To be included, FSH levels had to be above 30 U/L and estradiol levels had to be below 30 pg/mL on two different occasions, at least two months apart. Type 2 diabetes and prediabetes were diagnosed based on the American Diabetes Association criteria [49]. Subclinical hypothyroidism was defined as circulating TSH levels between 4.5 and 10.0 mU/L coexisting with free thyroid hormone concentrations within normal limits (free thyroxine between 10.0 and 21.2 pmol/L and free triiodothyronine between 2.2 and 6.5 pmol/L).

Women with monomeric hyperprolactinemia, other endocrine disorders, autoimmune or chronic inflammatory disorders, malabsorption or maldigestion, cardiovascular disease, hepatic or renal impairment, and other serious disorders were not considered for enrollment. We also excluded women on hormone replacement therapy and patients taking hypoglycemic drugs, drugs affecting pituitary function or drugs with known interactions with metformin.

The study population consisted of two groups. Group A (the control group) included women with prolactin levels within the reference range (total prolactin between 5 and 25 ng/mL, monomeric prolactin between 3.5 and 23.5 ng/mL) on at least two occasions. In turn, group B included women with macroprolactinemia, the diagnostic criteria of which were as follows: (a) total prolactin concentration exceeding 50 ng/mL, (b) prolactin recovery of less than 40%, and (c) post-precipitation plasma levels of prolactin below 26 ng/mL.

### 4.3. Study Size

**Figure 5:**
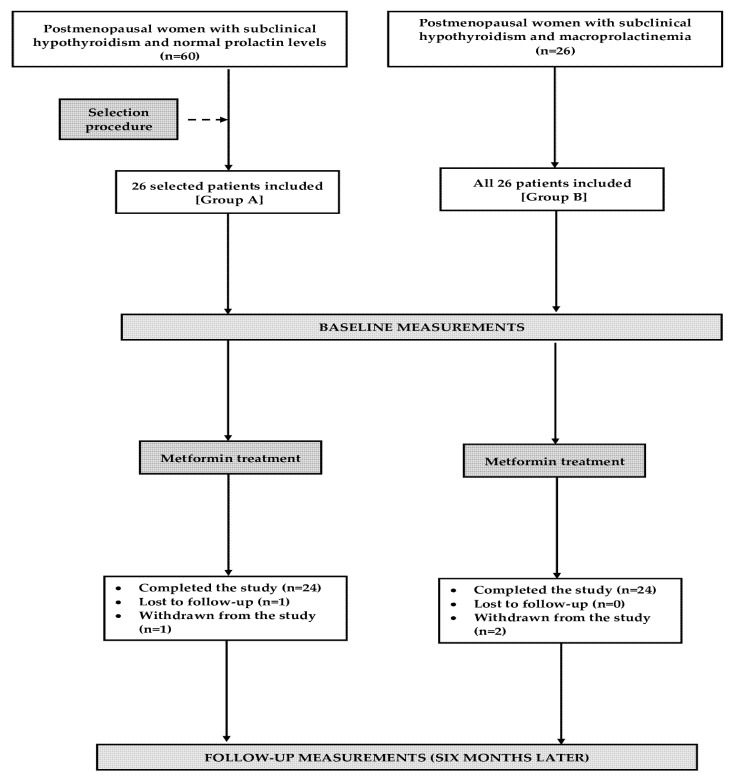
The flow of patients through the study.

Based on the sample size calculation, we estimated that at least 22 patients per group would be required to detect a 20% difference in mean concentrations of TSH and gonadotropins between both study groups, with a statistical power of 80% and significance level (α) of 0.05. To compensate for possible dropouts and losses to follow-up, the final sample size was increased by four patients per group. Only 26 out of 60 women with normal prolactin levels were included because our intention was to compare two groups showing no differences in terms of age, insulin sensitivity, and plasma concentrations of gonadotropins and TSH (Figure 5). The algorithm of this matching procedure was based on the minimum Euclidean distance rule. Considering possible variations in the outcome variables, similar proportions of women were included during the winter season (six in each group), spring season (seven in group A and six in group B), summer season (six in group A and seven in group B), and autumn season (seven in each group).

### 4.4. The Course of the Study

The study lasted for six months, and throughout this period of time, all the women were treated with metformin. The initial dose (0.5 g two times a day) was administered for 7 days, and then gradually (over 14–28 days) increased to 1 g three times a day. This final dose was used until the end of the study. To reduce adverse gastrointestinal effects, the drug was taken with meals or shortly after eating and swallowed whole with a glass of water. As before the study, all the participants were required to adhere to non-pharmacological interventions. Other chronic treatments were not allowed during the study. Acetaminophen, non-steroidal anti-inflammatory drugs, antimicrobials, antitussives, laxatives, antidiarrheal drugs or sleep-inducing drugs were accepted if the treatment period did not exceed 10 days, and the treatment was discontinued at least four weeks before the final visit. Adherence to metformin treatment was evaluated through pill counts and participant self-reporting. Good compliance was defined as the taking of at least 90% of the prescribed tablets. The withdrawal criteria were as follows: serious adverse effects (defined according to the Food and Drug Administration criteria [50]), changes in pharmacological treatment (other than that mentioned above), poor treatment adherence, and consent withdrawal.

### 4.5. Outcome Variables and Measurements

All biochemical variables were determined before metformin treatment and six months later. Fasting venous blood samples were collected in the morning hours (7.00–8.30 a.m.) following a 12 h fast. The measurements were performed in duplicate by a person blinded to the experimental protocol. Because prolactin secretion is pulsatile [45] and increases in response to stress and venipuncture [51], its levels were assessed in three blood samples taken at 20 min intervals in a quiet room, and the obtained results were averaged. Fasting plasma glucose, whole blood content of glycated hemoglobin (HbA_1c_), and safety biochemical parameters were measured using the multi-analyzer COBAS Integra 400 Plus purchased from Roche Diagnostics (Basel, Switzerland). Insulin, FSH, LH, TSH, prolactin, estradiol, free thyroxine, and free triiodothyronine were measured by electrochemiluminescence immunoassays (ADVIA Centaur XP Immunoassay System, Siemens Healthcare Diagnostics, Munich, Germany). Monomeric prolactin was measured after isolation performed using the polyethylene glycol precipitation method [24]. Macroprolactin was calculated as the difference between total (before precipitation) and monomeric (after precipitation) prolactin. Adrenocorticotropic hormone (ACTH) and insulin-like growth factor-1 (IGF-1) were measured in a solid-phase, competitive immunoassay using enzyme-labelled ligand (Siemens Immulite, Munich, Germany). HOMA-IR, to estimate insulin sensitivity, was calculated by dividing the product of glucose (mg/dL) and insulin (mU/L) by 405 [52]. The estimated glomerular filtration rate was calculated by using a shortened version of the modification of diet in renal disease equation.

### 4.6. Statistical Analysis

Statistical calculations were carried out using the Statistica 12.0 PL software (StatSoft Poland, Cracow, Poland). Potential sources of bias were addressed by strict inclusion criteria, ensuring the sample’s relevance to the research context, fidelity to the study protocol, and avoidance of poor adherence and unintended co-interventions, in order to obtain complete data and allow all investigators to independently interpret the results. Quantitative variables were handled by recoding, calculations, and categorizations. All raw data were logarithmically transformed to achieve homogeneous variances. Time since menopause, smoking status, and seasonal hormonal fluctuations were considered potential confounders for adjustment. The study groups were compared using unpaired *t*-tests, while intra-group comparisons were performed using a paired-sample Student’s test. Categorical data were analyzed using the chi-square test. Pearson’s correlation coefficient was used to evaluate possible correlations between the studied variables. All *p*-values were corrected for multiple testing using the Benjamini–Hochberg procedure. Adjusted two-tailed *p*-values below 0.05 were considered significantly different.

## 5. Conclusions

Chronic metformin treatment reduces FSH and TSH levels in hypothyroid postmenopausal women with normal prolactin homeostasis, but both effects are absent in the case of concomitant macroprolactinemia. The inhibitory action on the pituitary effects of metformin is determined by the severity of macroprolactinemia. Differences in the impact on hormone levels between women with and without macroprolactin excess are paralleled by similar differences in the impact on glucose homeostasis, mainly on insulin sensitivity. It seems that macroprolactin interacts with metformin at the level of the overactive pituitary cells, either directly by cellular metabolic pathways, or indirectly by hypothalamic control of pituitary function. Because of the novelty of our findings and the study’s limitations, the obtained results ought to be validated by other research groups.

## Figures and Tables

**Table 1:** Baseline characteristics of women participating in the study (intention-to-treat analysis).

Variable	Group A	Group B	*p*-Value
Number (n)	26	26	-
Age (years)	65 ± 9	66 ± 8	0.6738
Smokers (%)/number of cigarettes a day (n)/duration of smoking (years)	42/10 ± 6/32 ± 16	46/9 ± 6/35 ± 17	0.6015
Diabetes/prediabetes (%)	54/46/50	54/46	1.0000
Body mass index (kg/m^2^)	25.0 ± 5.0	24.7 ± 5.1	0.8312
Systolic blood pressure (mmHg)	132 ± 15	130 ± 17	0.6548
Diastolic blood pressure (mmHg)	86 ± 8	85 ± 7	0.6440
Fasting glucose (mg/dL) [70–99]	120 ± 15	122 ± 16	0.6438
HbA_1c_ (%) [<5.7]	6.7 ± 0.5	6.7 ± 0.6	1.0000
HOMA1-IR [<2.0]	4.6 ± 1.9	4.4 ± 1.7	0.6908
FSH (U/L) [>30]	78 ± 25	82 ± 30	0.6038
LH (U/L) [>15]	49 ± 21	51 ± 23	0.7447
TSH (mU/L) [0.4–4.5]	7.5 ± 1.2	7.4 ± 1.3	0.7751
Total prolactin (ng/mL) [5–25]	15.0 ± 6.5	84.0 ± 21.3	**<0.0001**
Monomeric prolactin (ng/mL) [3.5–23.5]	12.8 ± 5.7	13.0 ± 6.0	0.9024
Macroprolactin (ng/mL) [<3.0]	2.2 ± 0.9	71.0 ± 20.8	**<0.0001**
ACTH (pg/mL) [14–70]	38 ± 15	36 ± 16	0.6440
Estradiol (pg/mL) [<30]	18 ± 6	17 ± 8	0.6124
Free thyroxine (pmol/L) [10.5–21.8]	13.8 ± 2.4	14.9 ± 2.8	0.1346
Free triiodothyronine (pmol/L) [2.2–6.6]	3.5 ± 0.7	3.4 ± 0.7	0.6088
IGF-1 (ng/mL) [75–180]	130 ± 34	128 ± 42	0.8511

Group A: postmenopausal women with subclinical hypothyroidism and normal prolactin levels; group B: postmenopausal women with subclinical hypothyroidism and macroprolactinemia. Unless otherwise stated, the data are shown as the mean ± standard deviation. Statistically significant changes have been marked in bold. Reference values have been provided in square brackets.

**Table 2:** The impact of metformin on the safety biochemical parameters and glucose (intention-to-treat analysis).

Variable	Group A	Group B	*p*-Value
**Alkaline Phosphatase** (U/L) [30–120]			
Before treatment	65 ± 20	71 ± 29	0.3894
After treatment/at dropout	68 ± 27	69 ± 31	0.9018
*p*-value (before vs. after treatment/at dropout)	0.6510	0.8111	-
**Alanine transaminase** (U/L) [<35]			
Before treatment	17 ± 12	15 ± 12	0.5506
After treatment/at dropout	20 ± 13	19 ± 14	0.7906
*p*-value (before vs. after treatment/at dropout)	0.3914	0.2740	-
**Aspartate transaminase** (U/L) [<35]			
Before treatment	21 ± 14	20 ± 12	0.7832
After treatment/at dropout	23 ± 12	25 ± 14	0.5827
*p*-value (before vs. after treatment/at dropout)	0.5827	0.1729	-
**Bilirubin** (μmol/L) [5–20]			
Before treatment	11 ± 4	12 ± 5	0.4296
After treatment/at dropout	11 ± 4	11 ± 3	1.0000
*p*-value (before vs. after treatment/at dropout)	1.0000	0.3860	-
**Creatine kinase [25–200]**			
Before treatment	72 ± 25	80 ± 28	0.2824
After treatment/at dropout	79 ± 26	88 ± 32	0.2710
*p*-value (before vs. after treatment/at dropout)	0.3271	0.3420	-
** Estimated glomerular filtration rate ** (mL/min/1.73 m^2^) [>90]			
Before treatment	93 ± 16	92 ± 15	0.8110
After treatment/at dropout	91 ± 14	94 ± 17	0.4905
*p*-value (before vs. after treatment/at dropout)	0.6340	0.6548	-
**Glucose** (mg/dL) [70–99]			
Before treatment	120 ± 15	122 ± 16	0.6440
After treatment/at dropout	100 ± 12	109 ± 13	** 0.0124 **
*p*-value (before vs. after treatment/at dropout)	**<0.0001**	**0.0023**	-

Group A: postmenopausal women with subclinical hypothyroidism and normal prolactin levels; group B: postmenopausal women with subclinical hypothyroidism and macroprolactinemia. The data are shown as the mean ± standard deviation. Statistically significant changes have been marked in bold. Reference values have been provided in square brackets.

## Data Availability

The data that support the findings of this study are available from the corresponding author upon reasonable request.

## Supplementary Materials

The following supporting information can be downloaded at: https://www.mdpi.com/article/10.3390/ph18060834/s1, Table S1. The impact of metformin on the outcome variables in both groups of patients; Table S2. Percentage changes from baseline in the outcome variables in the study population; Table S3. STROBE Statement—Checklist of items that should be included in reports of case-control studies.
