# Supplementary material for: The Effect of Metformin on Pituitary Function in Postmenopausal Women with Subclinical Hypothyroidism and Macroprolactinemia: A Single-Center Prospective Case–Control Study
Source: Pharmaceuticals (Basel). 2025 Jun 2;18(6):834. doi: 10.3390/ph18060834 (PMC12195766; doi:10.3390/ph18060834)
Supplement: Supplementary file 1 [file pharmaceuticals-18-00834-s001.zip › pharmaceuticals-3642201-supplementary.pdf]

Table S1. The impact of metformin on the outcome variables in both groups of patients.

Variable	Group A	Group B	*p*-value
Fasting glucose (mg/dL) [70-99]			
Before treatment	119±12	121±14	0.5977
After treatment	98±10	107±12	**0.0070**
p-value (before *vs*. after treatment)	**<0.0001**	**0.0005**	-
HbA_{1c} [<5.7]			
Before treatment	6.6±0.5	6.7±0.5	0.4919
After treatment	5.5±0.6	6.0±0.5	**0.0030**
p-value (before *vs*. after treatment)	**<0.0001**	**<0.0001**	-
HOMA1-IR [<2.0]			
Before treatment	4.4±1.8	4.2±1.5	0.6778
After treatment	2.1±0.9	3.0±1.2	**0.0051**
p-value (before *vs*. after treatment)	**<0.0001**	**0.0037**	-
FSH (U/L) [>30]			
Before treatment	75±23	80±29	0.5114
After treatment	51±18	72±25	**0.0017**
p-value (before *vs*. after treatment)	**0.0002**	0.3114	-
LH (U/L) [>15]			
Before treatment	48±20	50±23	0.7493
After treatment	37±19	48±17	**0.0040**
p-value (before *vs*. after treatment)	0.0569	0.7440	
TSH (mU/L) [0.4-4.5]			
Before treatment	7.4±1.4	7.2±1.6	0.6471
After treatment	5.5±1.7	6.7±1.8	**0.0218**
p-value (before *vs*. after treatment)	**0.0001**	0.3144	-
Total prolactin (ng/mL) [5-25]			
Before treatment	15.2±6.3	83.2±21.5	**<0.0001**
After treatment	14.8±5.9	85.4±23.4	**<0.0001**
p-value (before *vs*. after treatment)	0.8214	0.7360	-
Monomeric prolactin (ng/mL) [3.5-23.5]			
Before treatment	13.2±6.1	12.8±5.8	0.8168
After treatment	12.6±5.5	13.0±5.0	0.7932
p-value (before *vs*. after treatment)	0.7221	0.8987	-

Macroprolactin (ng/mL) [<3.0]			
Before treatment	2.0±1.0	70.4±20.6	**<0.0001**
After treatment	2.2±1.0	72.4±23.0	**<0.0001**
p-value (before *vs*. after treatment)	0.4918	0.7525	-
ACTH (pg/mL) [14-70]			
Before treatment	40±16	37±18	0.5447
After treatment	46±20	47±21	0.8667
p-value (before *vs*. after treatment)	0.2571	0.2763	-
Estradiol (pg/mL) [<30]			
Before treatment	17±7	18±6	0.5978
After treatment	19±6	18±5	0.5440
p-value (before *vs*. after treatment)	0.2934	1.0000	-
Free thyroxine (pmol/L) [10.5-21.8]			
Before treatment	13.9±2.3	14.7±2.6	0.2647
After treatment	15.0±2.5	15.5±3.0	0.5340
p-value (before *vs*. after treatment)	0.1195	0.3287	-
Free triiodothyronine (pmol/L) [2.2-6.6]			
Before treatment	3.6±0.7	3.5±0.6	0.5977
After treatment	3.7±0.8	3.7±0.7	1.0000
p-value (before *vs*. after treatment)	0.6471	0.2934	-
IGF-1 (ng/mL) [75-180]			
Before treatment	126±32	131±40	0.6348
After treatment	138±41	145±43	0.5664
p-value (before *vs*. after treatment)	0.2642	0.2489	-

Group A: postmenopausal women with subclinical hypothyroidism and normal prolactin levels; Group B: postmenopausal women with subclinical hypothyroidism and macroprolactinemia; The data are shown as the mean ± standard deviation. Only women who completed the study were analyzed. Statistically significant changes have been marked in bold.

Table S2. Percentage changes from baseline in the outcome variables in the study population.

Variable	Group A	Group B	*p*-value
Δ Fasting glucose	-18±8	-12±6	**0.0051**
Δ HbA_{1c}	-17±8	-10±8	**0.0040**
Δ HOMA1-IR	-52±29	-29±26	**0.0058**
Δ FSH	-32±19	-10±19	**0.0002**
Δ LH	-23±28	-4±26	**0.0188**
Δ TSH	-26±16	-7±12	**<0.0001**
Δ Total prolactin	-3±14	2±12	0.4295
Δ Monomeric prolactin	-5±20	2±15	0.1768
Δ Macroprolactin	10±20	3±18	0.2089
Δ ACTH	15±29	27±32	0.1801
Δ Estradiol	12±28	0±25	0.1242
Δ Free thyroxine	8±11	5±8	0.2855
Δ Free triiodothyronine	3±8	6±10	0.2571
Δ IGF-1	10±20	11±25	0.8971

Group A: postmenopausal women with subclinical hypothyroidism and normal prolactin levels; Group B: postmenopausal women with subclinical hypothyroidism and macroprolactinemia; The data are shown as the mean ± standard deviation. Only women who completed the study were analyzed. Statistically significant changes have been marked in bold.

Table S3. STROBE Statement—Checklist of items that should be included in reports of case-control studies.

Section/Topic	Item #	Recommendation	Reported on page #
**Title and abstract**	1	(*a*) Indicate the study's design with a commonly used term in the title or the abstract	1
		(*b*) Provide in the abstract an informative and balanced summary of what was done and what was found	1
**Introduction**			
Background/rationale	2	Explain the scientific background and rationale for the investigation being reported	1-2
Objectives	3	State specific objectives, including any prespecified hypotheses	2, Figure 1
**Methods**			
Study design	4	Present key elements of study design early in the paper	11-12 [section 4.1]
Setting	5	Describe the setting, locations, and relevant dates, including periods of recruitment, exposure, follow-up, and data collection	11-12 [section 4.1]
Participants	6	(*a*) Give the eligibility criteria, and the sources and methods of case ascertainment and control selection. Give the rationale for the choice of cases and controls	12 [section 4.2]
		(*b*) For matched studies, give matching criteria and the number of controls per case	12 [section 4.3]
Variables	7	Clearly define all outcomes, exposures, predictors, potential confounders, and effect modifiers. Give diagnostic criteria, if applicable	14 [section 4.5]
Data sources/ measurement	8*	For each variable of interest, give sources of data and details of methods of assessment (measurement). Describe comparability of assessment methods if there is more than one group	14 [section 4.5]
Bias	9	Describe any efforts to address potential sources of bias	14 [section 4.6]
Study size	10	Explain how the study size was arrived at	12 [section 4.3]
Quantitative variables	11	Explain how quantitative variables were handled in the analyses. If applicable, describe which groupings were chosen and why	14 [section 4.6]
Statistical methods	12	(*a*) Describe all statistical methods, including those used to control for confounding	14 [section 4.6]
		(*b*) Describe any methods used to examine subgroups and interactions	14 [section 4.6]
		(*c*) Explain how missing data were addressed	N/A
		(*d*) If applicable, explain how matching of cases and controls was addressed	14 [section 4.6]
		(*e*) Describe any sensitivity analyses	14 [section 4.6]
**Results**			

Participants	13*	(a) Report numbers of individuals at each stage of study—eg numbers potentially eligible, examined for eligibility, confirmed eligible, included in the study, completing follow-up, and analysed	13 [Figure 5]
		(b) Give reasons for non-participation at each stage	3, 13 [Figure 5]
		(c) Consider use of a flow diagram	13 [Figure 5]
Descriptive data	14*	(a) Give characteristics of study participants (eg demographic, clinical, social) and information on exposures and potential confounders	4 [Table 1]
		(b) Indicate number of participants with missing data for each variable of interest	N/A
Outcome data	15*	Report numbers in each exposure category, or summary measures of exposure	3-8
Main results	16	(*a*) Give unadjusted estimates and, if applicable, confounder-adjusted estimates and their precision (eg, 95% confidence interval). Make clear which confounders were adjusted for and why they were included	4-5, 14, Supplementary Tables S1 and S2
		(*b*) Report category boundaries when continuous variables were categorized	N/A
		(*c*) If relevant, consider translating estimates of relative risk into absolute risk for a meaningful time period	N/A
Other analyses	17	Report other analyses done—eg analyses of subgroups and interactions, and sensitivity analyses	N/A
**Discussion**			
Key results	18	Summarise key results with reference to study objectives	8-9
Limitations	19	Discuss limitations of the study, taking into account sources of potential bias or imprecision. Discuss both direction and magnitude of any potential bias	11
Interpretation	20	Give a cautious overall interpretation of results considering objectives, limitations, multiplicity of analyses, results from similar studies, and other relevant evidence	10-11
Generalisability	21	Discuss the generalisability (external validity) of the study results	9-10
**Other information**			
Funding	22	Give the source of funding and the role of the funders for the present study and, if applicable, for the original study on which the present article is based	15

*Give information separately for cases and controls.

Note: An Explanation and Elaboration article discusses each checklist item and gives methodological background and published examples of transparent reporting. The STROBE checklist is best used in conjunction with this article (freely available on the Web sites of PLoS Medicine at http://www.plosmedicine.org/, Annals of Internal Medicine at http://www.annals.org/, and Epidemiology at http://www.epidem.com/). Information on the STROBE Initiative is available at www.strobe-statement.org.
